# Blink and you’ll miss it: the role of blinking in the perception of magic tricks

**DOI:** 10.7717/peerj.1873

**Published:** 2016-04-04

**Authors:** Richard J. Wiseman, Tamami Nakano

**Affiliations:** 1 Psychology, University of Hertfordshire, Hatfield, United Kingdom; 2 Graduate School of Frontier Biosciences, Osaka University, Suita, Osaka, Japan

**Keywords:** Psychology, Perception, Blinking, Magic, Attention, Illusion, Conjuring

## Abstract

Magicians use several techniques to deceive their audiences, including, for example, the misdirection of attention and verbal suggestion. We explored another potential stratagem, namely the relaxation of attention. Participants watched a video of a highly skilled magician whilst having their eye-blinks recorded. The timing of spontaneous eye-blinks was highly synchronized across participants. In addition, the synchronized blinks frequency occurred immediately after a seemingly impossible feat, and often coincided with actions that the magician wanted to conceal from the audience. Given that blinking is associated with the relaxation of attention, these findings suggest that blinking plays an important role in the perception of magic, and that magicians may utilize blinking and the relaxation of attention to hide certain secret actions.

## Introduction

Psychologists have long been interested in why audiences are fooled by magic tricks (see, e.g., Binet, 1894; Jastrow, 1896: Triplett, 1900). During a magic trick magicians attempt to hide the secret of their illusion (referred to as the ‘method’) and appear to perform a seemingly impossible feat (referred to as the ‘effect’). Most previous research has focused on the psychology that might underpin the efficacy of various methods (for a review of this work, see, e.g., Kuhn, Amlani & Rensink, 2008; Macknik, Martinez-Conde & Blakeslee, 2010; Thomas et al., 2015; Rensink & Kuhn, 2015), including the misdirection of attention (Kuhn & Martinez, 2011), verbal suggestion (Wiseman & Greening, 2005), and the disruption of problem solving (Danek et al., 2014).

However, researchers have yet to study one of the most frequently used attentional stratagems employed by magicians; namely, encouraging an audience to relax the intensity of their externalized attention whilst a secret action is being performed (Thomas et al., 2015). This omission is perhaps surprising, given that magicians have produced an extensive literature describing various techniques that encourage such relaxation of externalized attention. For example, Slydini (Ganson, 2001) demonstrated how magicians’ bodily movements can deceive audiences into thinking that nothing important is happening at certain moments, Kurtz (1998) discussed how performances often have a natural rhythm and how audiences relax their externalized attention on the ‘off-beats’ of this rhythm, De Ascanio (1964–2005) described how magicians’ ‘patter’ (the words spoken during a trick) can form a narrative that creates moments of relaxed external attention, and several writers (see, e.g., Lamont & Wiseman, 1999; Kuhn & Martinez, 2011; Kuhn et al., 2014) have outlined how making an audience laugh or applaud can also result in a relaxation of their externalized attention. The present study addresses this issue by exploring this stratagem.

Unfortunately, it is problematic to obtain an ongoing, continuous, unobtrusive and real-time measure of the degree to which an individual is attending to their surroundings. Fortunately, recent research suggests that the intensity of attention is highly correlated with a behaviour that is relatively straightforward to monitor and measure, namely, blinking. Blinking has been studied in a wide variety of contexts, including reading (Hall, 1945), face-to-face conversations (Nakano & Kitazawa, 2010),and the observation of videos (Nakano et al., 2009). This work demonstrated that the timing of participants’ eye-blinks are both highly synchronized and strongly associated with specific stimuli, such as full stops at the end of sentences, pauses in conversations, and the conclusion of narrative sequences during films. Several researchers have used these findings to speculate that blinking is associated with the relaxation of attention, and may represent a moment of ‘wakeful rest’ that helps boost the intensity of forthcoming attention (Ben-Simon et al., 2013). This notion has recently received direct support from an fMRI study showing that blinking precedes the deactivation of neural networks associated with attending to external stimuli, and the activation of regions associated with more internally oriented focus (Nakano et al., 2013). The present study extends this work by exploring the relationship between blinking and the relaxation of attention in a novel and real-world context, namely the perception of a magic trick.

Several researchers have studied the relationship between blinking, illusion, and magic (for a review of this work, see Macknik et al., 2008). O’Regan et al. (2000) made large changes to images each time participants blinked and discovered that the participants frequently failed to detect the changes. Kuhn & Tatler (2005) examined whether participants failed to see a secret action in a magic trick (the dropping of an object prior to it seeming to disappear) because they were blinking at the crucial moment. The results didn’t support this notion, but the video stimuli used in the study was short (around 15 s), and at the time of the drop the performer was strongly misdirecting participants’ attention to an alternative location.

Two magicians watched a short video of a highly skilled illusionist and identified when the illusionist was carrying out specific actions that were vital to the secret of the trick (the method) and when the illusionist was performing a seemingly impossible feat (the effect). Participants then watched the same video whilst their eye-blinks were recorded. Based on the previous research into attention and blinking, we hypothesized that the method would tend to coincide with moments of relaxed attention and so be associated with blinking, and that the effect would represent moments of heighted attention and so be associated with a lack of blinking.

## Method

### Participants

20 healthy adults (10 male, 10 female; age 22–47 years) with normal or corrected-to-normal visual acuity took part in the study. The study was approved by the review board of Osaka University, and all participants gave written informed consent before participation.

### Experimental stimulus

The stimulus video contained the highly skilled American magician Teller performing his version of a well-known trick called the ‘Miser’s Dream.’ At the start of his performance Teller invites a spectator on stage and asks them to hold a glass jar. Teller then magically produces a series of large silver coins from the air and from the spectator’s clothing. Each of these coins is dropped into the glass jar. At the end of the trick the coins are placed into a large tank of water and magically transform into goldfish. The video lasted 124 s, and was chosen because it is performed silently, is widely regarded by magicians as a brilliantly structured piece of magic, and involves several moments of both method and effect. Two magicians independently identified the times at which Teller secretly picked up the coins from a hidden location (referred to as ‘secret actions’), and when Teller performed a seemingly impossible effect (producing a series of coins or transforming the coins into fish). The experts agreed that there were seven secret actions and six effects. The two raters independently identified the frame numbers associated with both the start and end points of the secret actions and effects, with the data showing strong inter-rater agreement for both the secret actions and effects (Start frame: *N* = 13, *r* = .94, *p*(2 − *t*) < .0001; End frame: *N* = 13, *r* = .90, *p*(2 − *t*) < .0001).

### Experimental set up

The video (720 × 480 pixel) was presented on a 23-inch liquid crystal display (1,920 × 1,080 pixel; Dell, Plano, TX, USA). Participants placed their chin on the chinrest in front of the screen, which was 65 cm in front of the screen. The pupil size was recorded using a near-infrared eye tracking system (Eyelink; SR Research, Oakville, Ontario, CAN) with a sampling rate of 1 kHz while freely viewing the video. Participants were not informed that their blinking was being measured. All analyses of the eye-blink data were carried out using MATLAB.

## Results

Each eye-blink was automatically detected according to two criteria, namely that participants’ pupil size changed to zero for 20–500 ms. Each onset time was subsequently confirmed individually by the experimenter. The mean blink rate across participants while viewing the video was 15.4 min^−1^ (S.D. 10.6; range 3.4–36.3). The mean blink rate in females was slightly higher than that in males (female 17.5 min^−1^: male 13.3 min^−1^), but this difference was not significant (t_9_ = 1.2, *p* = 0.3).

To discover whether blinking was synchronized across participants, we analyzed the distribution of blink onset asynchrony for all combinations of two pairs with a bin width of 300 ms (see Nakano et al., 2009). This bin width was selected because the peak at zero time point was the steepest. For each combination of participants we assigned a reference to one participant and a test to another. We then calculated the onset asynchrony of all eyeblinks in the test participant from each eyeblink in the reference participant. The histograms of the onset asynchrony was normalized by the total number of eyeblinks among the reference participants. Thus, each participant had nineteen combinations with the other participants.

The histograms of the onset asynchrony was normalized by the total number of eye-blinks of each participant. We then created 1,000 surrogate histograms of blink onset asynchrony for each combination of two pairs by shuffling the inter-blink intervals (IBIs) of the original data. The randomized time series preserved the exact distribution of IBIs but lost the timing structure. The count in each bin of the original histogram was then transformed into a *Z*-score by using the mean and the standard deviation of the counts in the 1,000 randomized histograms. We applied one sample *t*-tests to the *Z* scores from all combinations for each of 13 bins to see whether the blinks of participants were significantly synchronized. Bonferroni corrections were applied to the multiple comparisons. To characterize each frame of synchronous blinks, we counted how many participants blinked at each video frame.

**Figure 1:**
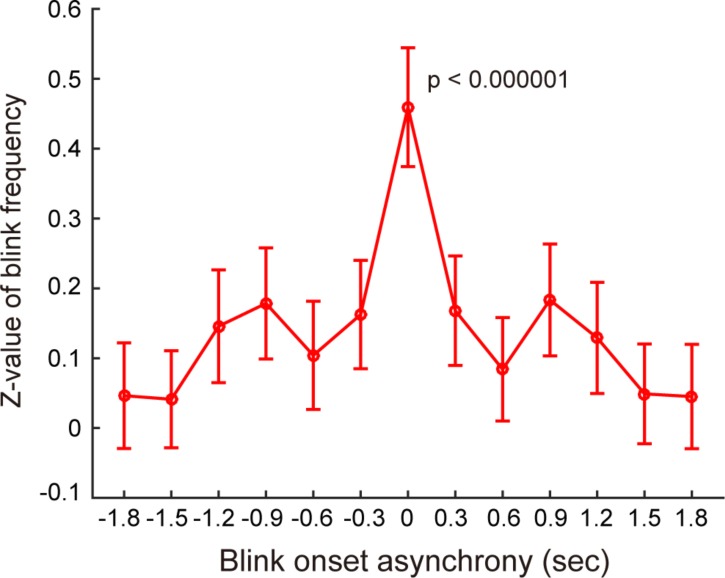
Distributions of eyeblink onset asynchrony across participants. The blink frequency was transformed into *Z* score using a distribution of 1,000 randomized surrogate data. Error bars represent the standard errors among all combinations of two pairs.

**Figure 2:**
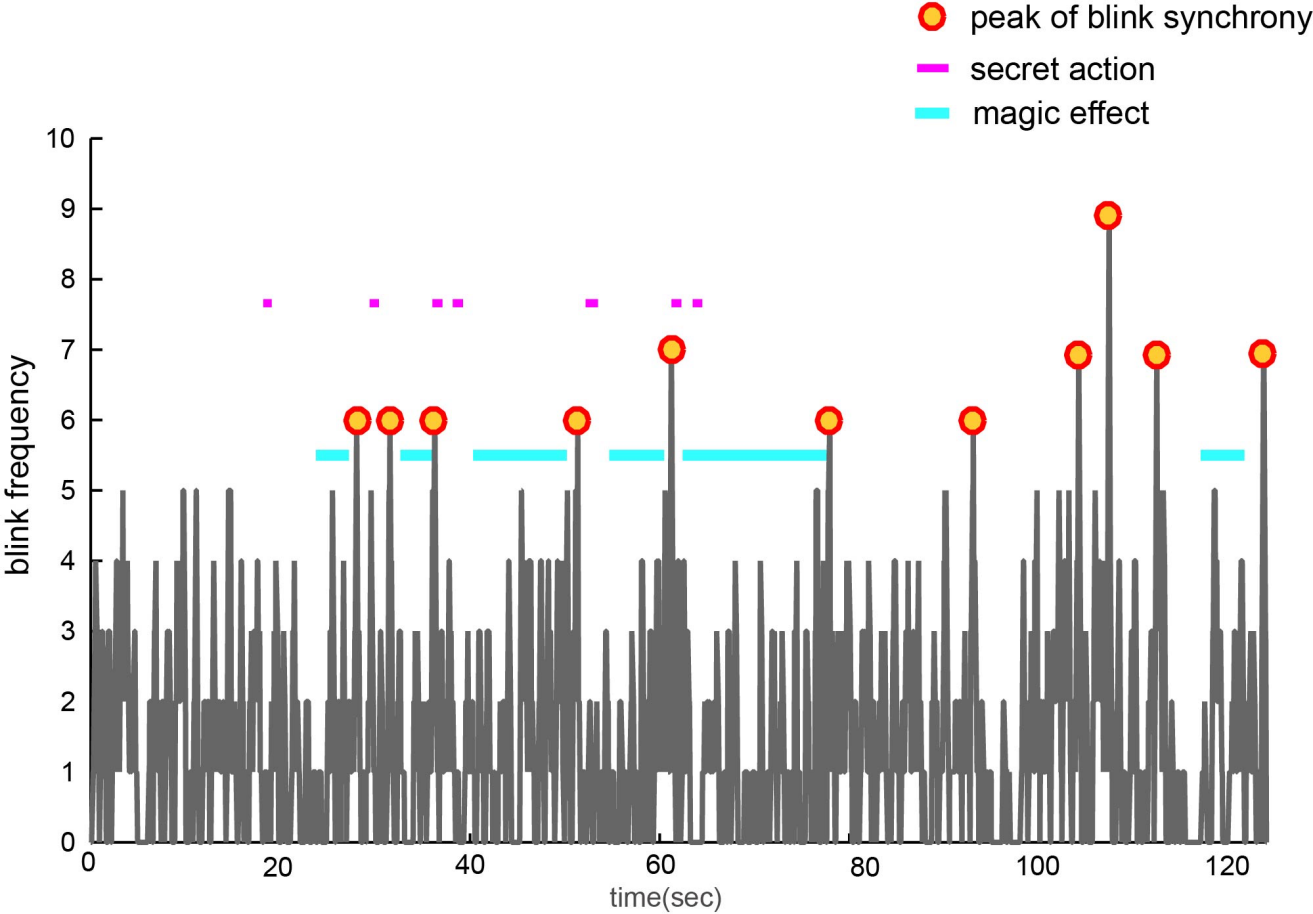
Timeline of the video, showing frequency histograms of eye-blinks, synchronized blinks, secret actions and magical effects.

We examined whether the blinks synchronize across participants while viewing the video by comparing the original data with the randomized surrogate data. The distribution of the blink onset asynchrony showed a prominent peak in the 300 ms bin around zero (±150 ms, Fig. 1). This peak is significantly higher than 0 (*t*_379_ = 7.6, *p* < 0.000001). Comparison with the surrogate histogram created by the random shuffling of the IBIs revealed 11 moments when six or more participants blinked at the same time, which was significantly higher than random probability (*z* test, *p* < 0.0002; see Fig. 2).

The timings of the synchronized blinking, secret actions, and magical effects are shown in Fig. 2. Four of the seven secret actions were closely associated with a synchronized blink. In contrast, the synchronized blinks tended not to be associated with the effect periods, despite those periods lasting much longer than the secret actions. In addition, synchronized blinking frequently occurred immediately after the conclusion of a magical effect. Previous research into blinking during the observation of films suggests that blinks are not systematically associated with editing cuts in the film (Smith & Henderson, 2008; Nakano et al., 2009). This was also the case in the pre sent work, with the timing of the synchronized blinks being unrelated to the cuts in the stimulus video. The frame numbers associated with the synchronized blinks, cuts, secret actions and effects are supplied as Supplemental Information 3.

## Discussion

Participants’ eye-blinks were monitored while they watched a video of a highly skilled illusionist. Their eye-blinks were highly synchronized, thus providing additional support for the notion that blinking is not simply associated with the lubrication of the eye, but rather also serves a psychological function.

Two magicians had identified several moments when the illusionist carried out a secret action. It was hypothesized that these actions would be associated with a relaxation of the participants’ attention, and that they would therefore be likely to be blinking when the actions were carried out. This hypothesis was supported. In addition, it was predicted that the participants would tend not to blink when the magician was performing a seemingly impossible feat. Again, this hypothesis was supported as the synchronized blinks were not associated with the effect periods, despite those periods being significantly longer than the secret actions. These findings may prove beneficial to both psychologists and magicians.

This exploratory study presented an unusual and naturalistic test of the notion that blinking is associated with a relaxation of attention, and the positive results provide additional support for the theory. Magicians have developed a range of techniques that are designed to encourage an audience to relax their attention, and future work could provide additional insights into attention by employing this paradigm to systematically assess the efficacy of these techniques. Additional work could also explore the mechanism underlying the effect. It might be, for example, that when audiences blink, they are focusing their attention internally rather than externally, or that the blinks are themselves disrupt perception (e.g., both Costela et al. (2014) and Troncoso et al. (2015) have found that blinking leads to corrective microsaccades that are associated with suppressed vision).

In addition, these findings are likely to be of interest to magicians, and may even inform the performance of magic. Magicians have produced an extensive literature in the manipulation of externalized attention (see, e.g., Fitzkee, 1945; Tamariz, 2007; Ortiz, 2006). Although magicians understand the importance of performing secret actions when an audience relaxes their externalized attention, they have no easy way of discovering when such moments occur. Similarly, they appreciate the importance of ensuring that audiences are attending at the moment of effect, but again, have no real way of knowing if this happening during a specific performance. The current technique provides an unobtrusive and real time measure of attention, and so could be used to identify moments when audiences are, and are not, attending to their surroundings. The resulting data could then be used to modify performances to maximally exploit these moments.

Although the results are positive, the stimulus video was specifically chosen in the hope of obtaining the effect. The illusionist involved is highly skilled and extremely experienced, and the trick itself represents a certain type of performance (for example, it has several phrases, carried out in silence, and performed on stage). As such, the study demonstrates how this novel paradigm can be used to explore blinking and attention, and provides grounds for thinking that blinking may play an important, and hitherto unrecognized, role in magic. However, it is important that future studies employ a range of video stimuli to discover whether the effect is also occurs with other magicians, tricks, and performances.

In conclusion, this study has uncovered a novel and previously unknown relationship between blinking and the performance of magic, and it is hoped that the future exploration of this effect will benefit both psychologists and magicians.

## Supplemental Information

10.7717/peerj.1873/supp-1 Supplemental Information 1 Code

10.7717/peerj.1873/supp-2 Supplemental Information 2 Blinking data

10.7717/peerj.1873/supp-3 Supplemental Information 3 Frame numbers for secret actions, blinks, effects and cuts

## Additional Information and Declarations

### Data Availability

The following information was supplied regarding data availability: The raw data has been supplied as Supplemental Information.
