# Supplementary material for: Blink and you’ll miss it: the role of blinking in the perception of magic tricks
Source: PeerJ. 2016 Apr 4;4:e1873. doi: 10.7717/peerj.1873 (PMC4824881; doi:10.7717/peerj.1873)
Supplement: Supplemental Information 3 [file peerj-04-1873-s003.doc]

| **Frame numbers of synchronized blinks:**  841, 946, 1088, 1541, 1836, 2338, 2792, 3124, 3216, 3372, 3708 |
| --- |
| **Frame numbers of cuts:**  62, 720, 115, 175, 226, 383, 432, 527, 574, 605,633, 720, 829, 1002, 1063, 1111, 1228, 1284, 1364, 1431, 1490, 1664, 1735, 1801, 1839, 1934, 1972, 2018, 2127, 2299, 2356, 2630, 2680, 2720, 3260, 3353, 3595, 3661 |
| **Frame numbers of secret actions:**  621-622, 927-930, 1107-1113, 1166-1172, 1548-1560, 1795-1800, 1856-1860 |
| **Frame numbers of effects:**  730-800, 967-1068, 1227-1489, 1658-1797, 1890-2315, 3529-3633. |
